# Submucosal Lipoma-Induced Small Bowel Intussusception: A Case Study of Surgical Intervention and Management in an Adult Female

**DOI:** 10.7759/cureus.62710

**Published:** 2024-06-19

**Authors:** Kesav Sudabattula, Rajesh Gattani, Tejaswini Panchagnula, Tushar Dahmiwal, Nikhil Thatipalli, Bhagyesh Sapkale

**Affiliations:** 1 General Surgery, Jawaharlal Nehru Medical College, Datta Meghe Institute of Higher Education and Research, Wardha, IND; 2 General Surgery, Sapthagiri Institute of Medical Sciences and Research Centre, Bengaluru, IND; 3 Medicine, Jawaharlal Nehru Medical College, Datta Meghe Institute of Higher Education and Research, Wardha, IND

**Keywords:** postoperative care, benign tumors, adult intussusception, ct imaging, surgical resection, ileoileal anastomosis, bowel obstruction, abdominal pain, small bowel intussusception, submucosal lipoma

## Abstract

We present an adult patient, a 39-year-old female, with chief complaints of pain in the umbilical region. The patient was further evaluated by radiological investigations and was diagnosed with small bowel intussusception caused by submucosal lipoma as the lead point. She had undergone ileal resection and anastomosis of the affected segment. The postoperative period was uncomplicated, and the patient continued with regular oral intake. The histopathological analysis revealed it to be adipose tissue with no features of atypia. This case shows the rare presentation of small bowel intussusception due to a submucosal lipoma. It emphasizes the significance of diagnostic imaging tools for diagnosis and the need for surgery for proper administration.

## Introduction

The benign adipose tissue growth from the intestinal submucosa is called submucosal lipoma [1]. An intestinal section that telescopes or invaginates into the lumen of an adjacent segment is known as intussusception [2]. The bowel segment that is affected (the intussusceptum) telescopically or prolapsingly prolapses into another segment of the bowel (the intussuscipiens) due to the lead point being dragged forward by normal peristalsis in intussusception. Adhesions, polyps, viral infections, lipomas, Meckel's diverticulum, gastrointestinal stromal tumors, lymphomas, and other malignancies are among the etiologies of the lead point of intussusception. Telescoping of one portion of the ileum into another is known as ileoileal intussusception, which is less common compared to ileocecal intussusception [3]. A portion of the colon goes into its other portion, a condition called colonic intussusception [4]. The ileum goes into the colon, but it includes a larger range of the colon and extends past the cecum, a condition known as ileocolic intussusception [5].

Ileoileal intussusception is a medical condition in which a segment of the ileum (the final section of the small intestine) telescopes into another segment of the ileum, which is common in infancy [5,6]. A surgical operation known as ileoileal anastomosis joins together two ends of the ileum, the ultimate portion of the small intestine [6]. The anastomosis usually ensures that the intestinal contents may flow through the digestive tract [6]. While the lead point of small bowel intussusception is of malignant origin in 25% of cases and up to 66% of cases for large bowel intussusception, intussusception is not a common cause of small bowel obstruction in adults. As a result, it ought to be excluded right away and given special consideration. Intestinal ischemia, intestinal perforation, sepsis, shock, and peritonitis are just a few of the issues that intussusception can cause to the affected intestine segment, even if it is not cancerous. This is an uncommon instance of ileoileal intussusception caused by a submucosal lipoma [7].

## Case presentation

A 39-year-old female reported to the Emergency Medicine Department with chief complaints of abdominal pain in the periumbilical region for the previous three days, as well as several episodes of bilious vomiting. The patient characterized the discomfort as constant and intensifying. She reported no concurrent symptoms, such as fever, diarrhea, or blood in the stool. The patient had similar episodes three times, two months apart, for the past year, which were relieved on their own and did not seek any medical attention. During the physical examination, the patient seemed attentive and aware but was experiencing significant discomfort owing to abdominal pain.

**Figure 1:**
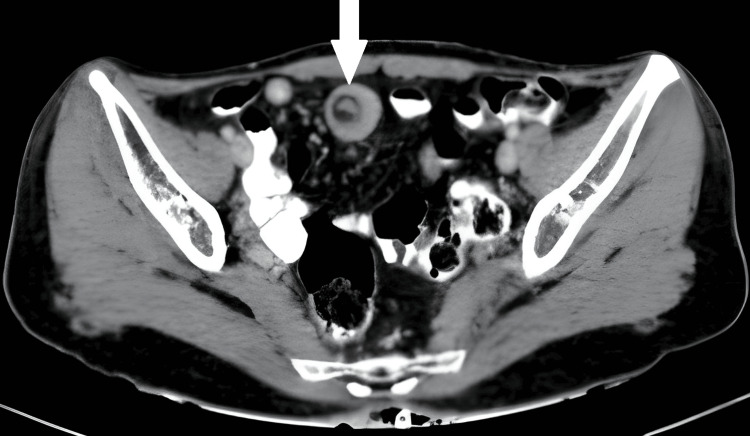
CT scan of the abdomen and pelvis The CT scan of the abdomen and pelvis showed a submucosal lipoma, coming about in small bowel intussusception and partial bowel obstruction, as indicated by the white arrow. CT: computed tomography.

The abdominal examination indicated diffuse tenderness in all four quadrants, with no guarding, stiffness, or distention. Auscultation revealed hypoactive bowel sounds. A digital rectal examination uncovered an empty, collapsed rectum without any discernible tumors. A computed tomography (CT) scan of the abdomen and pelvis revealed a submucosal lipoma in small bowel intussusception and partial bowel obstruction, as seen in Figure 1. Based on these results, the understanding of small bowel intussusception caused by a submucosal lipoma was analyzed.

**Figure 2:**
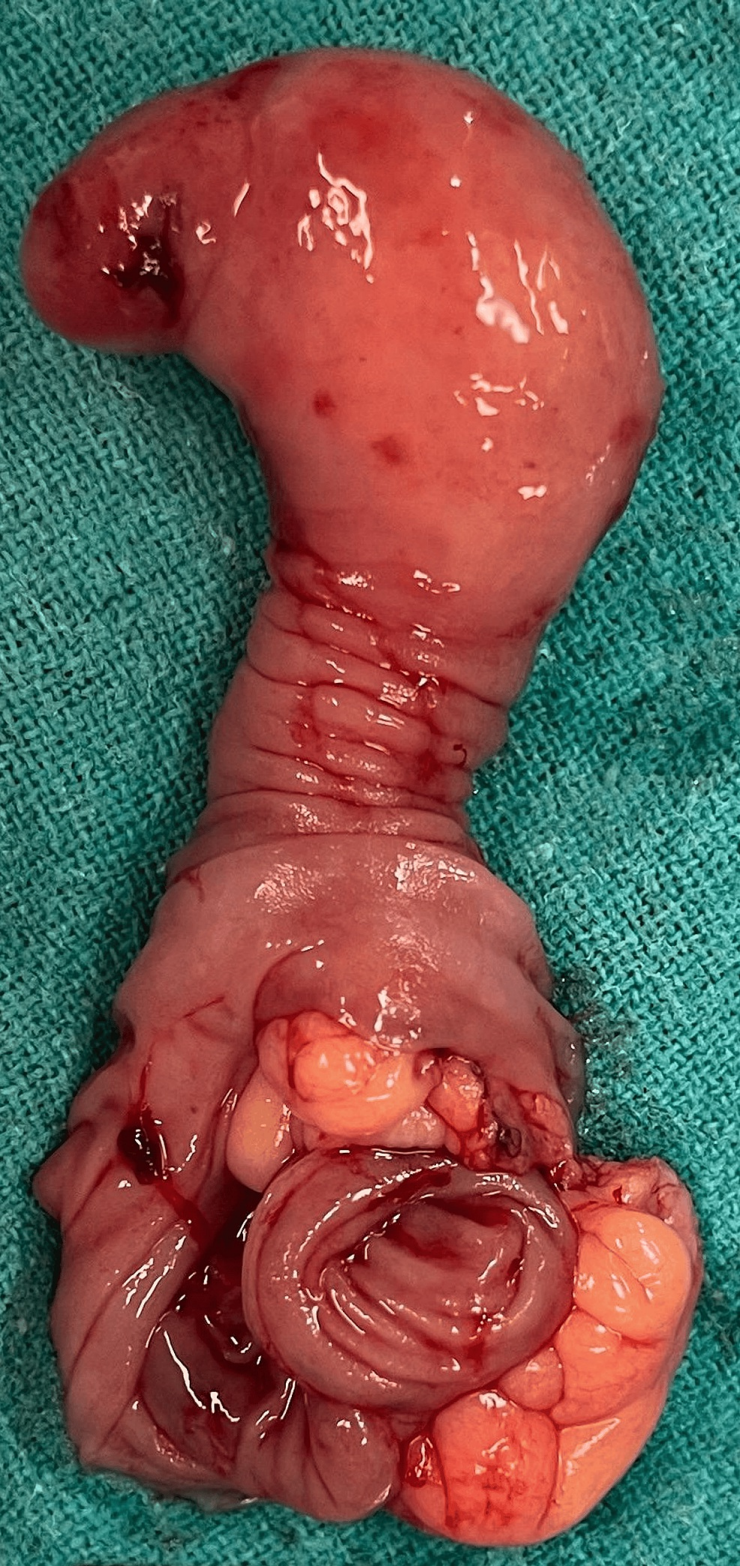
The excised ileal segment

The patient had undergone surgery, which included resection of the ileal segment with submucosal lipoma and an ileoileal anastomosis. A lower midline laparotomy incision was taken, and upon entering the abdominal cavity, the small bowel was examined for intussusception and the affected ileal segment. The next step was to isolate the affected ileal segment. Non-crushing clamps were used proximally and distally to the section to prevent bowel contents from spilling. The superior and inferior mesenteric vessels supplying the afflicted segment were located, ligated, and split to ensure that the remaining bowel received appropriate blood flow. This step was critical for avoiding unnecessary blood loss and ensuring the survival of the bowel. With the diseased ileal segment separated, transverse incisions were made through the bowel wall immediately beyond the clamps to excise the afflicted segment. The excised portion, comprising the submucosal lipoma, was subsequently removed from the surgical field, removing the cause of the intussusception. The excised ileal segment is shown in Figure 2.

**Figure 3:**
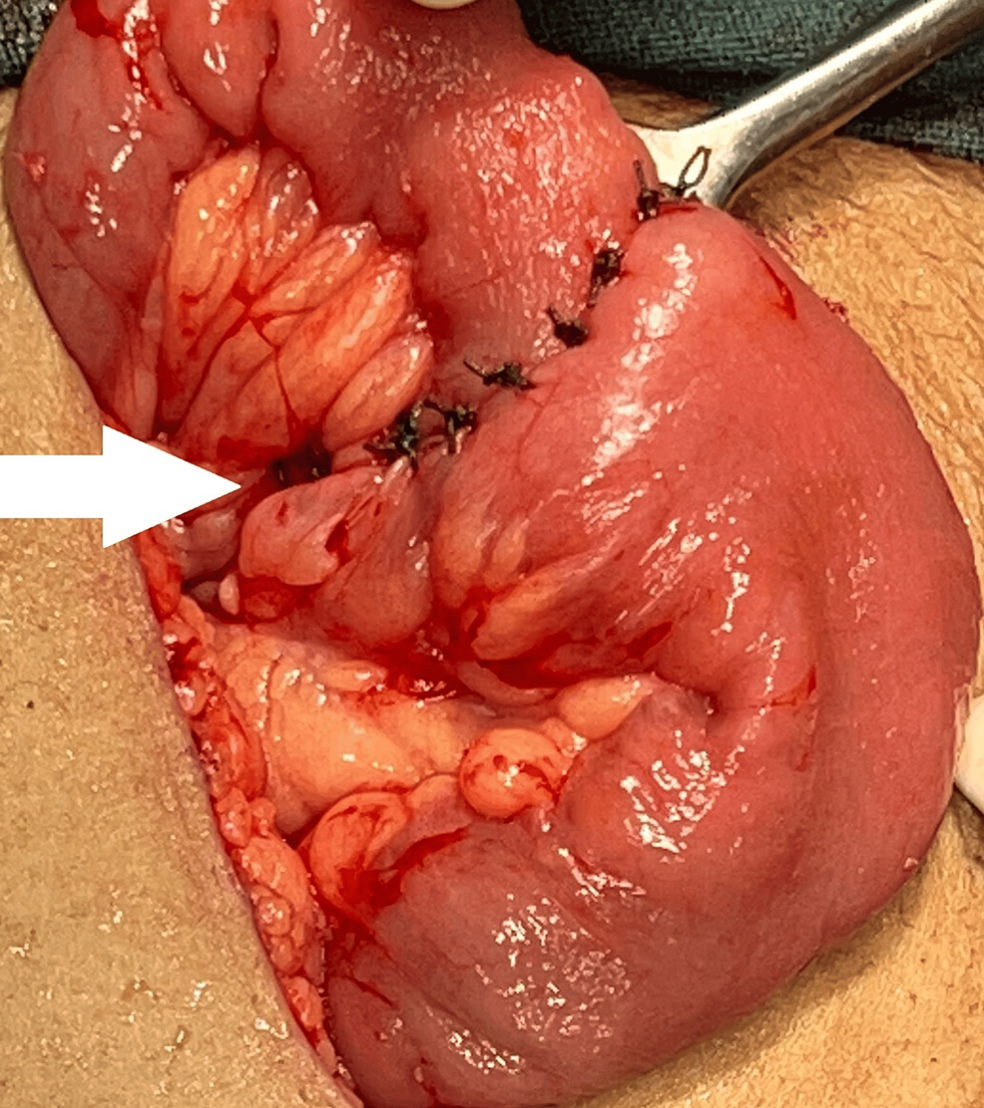
Ileoileal anastomosis The ileoileal anastomosis is indicated by the white arrow.

The bowel ends were then prepped for anastomosis by cutting uneven edges to achieve clean, straight edges. The two ends of the ileum were aligned before performing an end-to-end anastomosis with a hand-sewn technique. A two-layer approach was used in a hand-sewn anastomosis: the inner layer is closed with absorbable interrupted sutures to approximate the mucosa and submucosa. In contrast, the outside layer is closed with non-absorbable sutures to approximate the seromuscular layer. The ileoileal anastomosis is shown in Figure 3.

To confirm the integrity of the anastomosis, it was gently inflated with saline and tested for fluid leakage. Furthermore, the blood flow to the anastomosis was ensured for proper perfusion, which is necessary for healing. Saline was subsequently used to clean the abdominal cavity, removing any remaining blood and contaminants. Intra-abdominal drains were placed, and the laparotomy incision was closed in layers, with non-absorbable sutures closing the fascia, subcutaneous tissue being approximated as appropriate, and staples closing the skin.

Following surgery, the patient was observed in the recovery room for side effects of anesthesia and surgical issues. Postoperatively, the patient was started on broad-spectrum cephalosporin and nitroimidazole antibiotics. ERAS (enhanced recovery after surgery) protocol was followed postoperatively by a liquid diet followed by a soft diet on days two and three, respectively. On postoperative day two, the patient was mobilized out of bed and passed flatus. On day three, the patient passed stools, and abdominal drains were removed. The patient was discharged on day five, and skin suture removal was done on subsequent follow-up. The patient was followed up frequently for two years to monitor for recurrence.

In general, the case portrays the appearance, diagnosis, and compelling surgical treatment of small bowel intussusception caused by a submucosal lipoma in a 39-year-old female. The timeline of events in the given case is depicted in Table 1.

**Table 1:** Timeline of events The table was created by Kesav Sudabattula and Bhagyesh Sapkale.

Timeline	Event
Three days prior to admission	The patient experienced abdominal pain in the periumbilical region (chief complaint).
-	Episodes of bilious vomiting occur.
Admission to the Emergency Department	The patient reported abdominal pain and vomiting for the past 3 days (chief complaint).
-	Similar episodes occurred 3 times previously, 2 months apart, over the past year, and resolved on their own.
-	A physical exam revealed diffuse tenderness but no guarding, stiffness, or distention.
-	Hypoactive bowel sounds were detected.
-	The CT scan showed submucosal lipoma causing small bowel intussusception.
-	Diagnosis: small bowel intussusception caused by submucosal lipoma.
Surgery	The patient underwent surgery for resection of the affected ileal segment with submucosal lipoma and ileoileal anastomosis.
Preoperative preparation	The patient was positioned supine, given general anesthesia, and the surgical site was sterilized.
-	A midline laparotomy incision was made to access the abdominal cavity.
-	The small bowel was inspected to locate intussusception and the affected ileal segment.
-	The affected ileal segment was isolated with clamps placed proximally and distally.
-	The mesenteric vessels supplying the affected segment were located, ligated, and divided.
-	The affected ileal segment was resected, including the submucosal lipoma (cause of intussusception).
-	Bowel ends were prepped for anastomosis with clean, straight edges.
-	Ileal ends were aligned, and hand-sewn end-to-end anastomosis was performed (two-layer closure).
-	Anastomosis was tested for leaks and blood flow for proper perfusion.
-	The abdominal cavity was cleaned with saline.
-	Laparotomy incision closed in layers (fascia, subcutaneous tissue, skin).
Postoperative care	The patient was monitored in recovery for anesthetic recovery and surgical complications.
-	ERAS protocol, pain management, and gradual intake of oral feed were initiated.
Follow-up	The patient was monitored for 6 months with regular checkups.

## Discussion

The case of the 50-year-old male reported by Roy et al. presenting with acute abdominal pain shares several similarities with our case of a 39-year-old female who also presented with abdominal pain and vomiting [7]. Both patients exhibited side effects consistent with small bowel intussusception, a moderately uncommon condition in adults regularly accelerated by a lead point such as a submucosal lipoma. Both presented to the emergency department with diffuse abdominal pain and multiple episodes of non-bloody, non-bilious vomiting, with diffuse tenderness on physical examination but without signs of peritoneal irritation [8]. The CT imaging in both cases was essential in diagnosing the intussusception, uncovering a lead point caused by a submucosal lipoma, and showing bowel obstruction with edematous, dilated bowel but no free intraperitoneal air or fluid collection [8]. Surgery was required in both cases, including resection of the affected bowel segment followed by anastomosis [8]. Postoperative recovery was uneventful, with effective nasogastric tube removal on day two, progression to a soft diet on day three, flatus and stool passed on day three, and discharge from the hospital on day five.

In any case, there were outstanding differences between the cases. The male patient was 50 years old and had a history of psoriasis and hypertension. In contrast, the female was 39 years old with no comorbidity [8]. The male experienced symptoms for one day compared to three days in the female, possibly influencing the degree of bowel ischemia observed amid surgery. The specifics of the resected bowel segment, moreover, varied.

The male's resected bowel loop measured 13 x 5 cm with a connected mesentery of 13 x 3 x 2 cm and a 24 cm intussuscepted portion. At the same time, the female's case included a resected bowel loop measuring 6 x 5 cm, followed by ileoileal anastomosis, showing particular inclusion of the ileum [8]. Despite these contrasts, both cases uncovered comparative pathological findings of ischemic changes, ulceration, congestion, and submucosal lipoma at the lead point of intussusception, highlighting the part of submucosal lipomas in adult intussusception and the need for timely surgical intervention.

In comparing the case of a 68-year-old male detailed by Bokhari et al. with our case, both patients displayed symptoms demonstrative of bowel obstruction. However, the signs and diagnostic findings varied [8]. The 68-year-old male experienced extreme abdominal pain, nausea, vomiting, and constipation, with distention and tenderness within the right iliac fossa [8]. Both cases included radiologic imaging revealing bowel obstruction, with the male patient's CT scan certifying a small bowel obstruction due to ileocecal intussusception caused by intraluminal lipomas [8]. The fundamental pathology in both cases was benign lipomatous masses acting as the primary point for the intussusception.

Surgical intercession was required for both patients to resolve the obstruction and reduce symptoms. The male patient experienced an emergency laparotomy with resection of the cecum, appendix, and terminal ileum taken after a side-to-side anastomosis of the ileum to the ascending colon [8]. The histopathological investigation uncovered two pedunculated lipomas with focal ischemic hemorrhagic changes [8]. In addition, our female patient had the included ileal segment resected with an ileoileal anastomosis performed. In both cases, surgeries were effective, and postoperative recovery was uneventful. Eminently, the most distinction lies within the extent and location of the resections. The male patient's resection included the cecum and appendix, whereas the female's surgery was limited to the ileum [8]. Additionally, the histopathological discoveries were more complex within the male patient's case, uncovering ulcerative mucosa and ischemic changes compared to the simpler submucosal lipoma in our female patient.

In comparing the case of a 67-year-old male reported by Ahmed et al. with our case, both patients presented with abdominal pain and symptoms indicative of small bowel obstruction due to intussusception caused by submucosal lipomas [9]. The 67-year-old male experienced a five-day history of abdominal pain, nausea, bilious vomiting, and obstipation, with a past medical history of irritable bowel syndrome, hypertension, and an appendectomy [9]. His physical examination revealed a soft, distended abdomen, and a CT scan confirmed ileocecal intussusception [9]. The surgical intervention involved a laparoscopic right hemicolectomy with resection of the terminal ileum and an ileocolic anastomosis [9]. Gross pathology showed two submucosal lipomas and an ischemic terminal ileum loop, while histopathology confirmed the presence of mature adipose tissue consistent with a lipoma [9].

In differentiation, our 39-year-old female displayed a three-day history of periumbilical abdominal pain and multiple episodes of bilious vomiting. Her physical examination uncovered diffuse tenderness in all four quadrants without guarding, rigidity, or distention, and a digital rectal examination showed an empty, collapsed rectum. Comparable to the male patient, a CT scan distinguished small bowel intussusception caused by a submucosal lipoma [10]. The surgical strategy contrasted somewhat, including resectioning the affected ileal segment after an ileoileal anastomosis. Both cases included intussusception due to submucosal lipomas and required surgical resection. Still, the anatomical areas and types of anastomosis were diverse, reflecting the particular clinical scenarios and surgical approaches custom-fitted to each patient [10].

## Conclusions

This case highlights the uncommon event of submucosal lipoma-induced small bowel intussusception in a 39-year-old female, effectively managed with surgical resection and ileoileal anastomosis. Timely diagnosis and intercession are significant for favorable results, emphasizing the role of imaging and custom-made surgical approaches in managing adult intussusception due to benign tumors.

## References

[1] Lin YM, Chiu NC, Li AFY, Liu CA, Chou YH, Chiou YY (2017). Unusual gastric tumors and tumor-like lesions: radiological with pathological correlation and literature review. World J Gastroenterol.

[2] Marinis A, Yiallourou A, Samanides L, Dafnios N, Anastasopoulos G, Vassiliou I, Theodosopoulos T (2009). Intussusception of the bowel in adults: a review. World J Gastroenterol.

[3] Potts J, Al Samaraee A, El-Hakeem A (2014). Small bowel intussusception in adults. Ann R Coll Surg Engl.

[4] Lianos G, Xeropotamos N, Bali C, Baltoggiannis G, Ignatiadou E (2013). Adult bowel intussusception: presentation, location, etiology, diagnosis and treatment. G Chir.

[5] Lu T, Chng Y (2015). Adult intussusception. Perm J.

[6] Far SS, Miraj S (2016). Single-incision laparoscopy surgery: a systematic review. Electron Physician.

[7] Roy J, Sall K, Megaris A, DiRoma F, Mukherjee I (2021). Submucosal lipoma causing small bowel intussusception. Cureus.

[8] Bokhari SFH, Yaseen K, Abid S, Vohra RR, Sajid S (2022). Ileocecal intussusception with lipoma as a lead point leading to small bowel obstruction in an elderly male: a case report. Cureus.

[9] Ahmed M, Habis S, Saeed R, Mahmoud A, Plurad D (2018). Submucosal lipomas causing intussusception and small bowel obstruction: a case report. Cureus.

[10] Girish N, Thomas N, Natraj G, Kumar KVS, John N (2023). Unusual presentation of ileal intussusception due to submucosal lipoma in a child and its management. J Indian Assoc Pediatr Surg.

